# Coverage survey and lessons learned from a pre-emptive cholera vaccination campaign in urban and rural communities affected by landslides and floods in Freetown Sierra Leone

**DOI:** 10.1016/j.vaccine.2023.01.026

**Published:** 2023-03-31

**Authors:** Marcello Gelormini, Marissa Gripenberg, Dennis Marke, Mariama Murray, Sahr Yambasu, Mohamed Koblo Kamara, Caleb Michael Thomas, Kazungu Donald Sonne, Sibylle Sang, Janet Kayita, Lorenzo Pezzoli, Grazia Caleo

**Affiliations:** a World Health Organization, Geneva, Switzerland; b Médecins Sans Frontières, OCA, Amsterdam, the Netherlands; c Ministry of Health and Sanitation, Freetown, Sierra Leone; d Statistics Sierra Leone, Freetown, Sierra Leone; e World Health Organization, Freetown, Sierra Leone

**Keywords:** Cholera, Oral Cholera Vaccine, Sierra Leone, Vaccination Campaigns

## Abstract

**Background:**

On 14 August 2017, massive landslides and floods hit Freetown (Sierra Leone). More than 1,000 people lost their lives while approximately 6,000 people were displaced. The areas most affected included parts of the town with challenged access to basic water and sanitation facilities, with communal water sources likely contaminated by the disaster. To avert a possible cholera outbreak following this emergency, the Ministry of Health and Sanitation (MoHS), supported by the World Health Organization (WHO) and international partners, including Médecins Sans Frontières (MSF) and UNICEF, launched a two-dose pre-emptive vaccination campaign using Euvichol™, an oral cholera vaccine (OCV).

**Methods:**

We conducted a stratified cluster survey to estimate vaccination coverage during the OCV campaign and also monitor adverse events. The study population – subsequently stratified by age group and residence area type (urban/rural) – included all individuals aged 1 year or older, living in one of the 25 communities targeted for vaccination.

**Results:**

In total 3,115 households were visited, 7,189 individuals interviewed; 2,822 (39%) people in rural and 4,367 (61%) in urban areas. The two-dose vaccination coverage was 56% (95% confidence interval (CI): 51.0–61.5), 44% (95%CI: 35.2–53.0) in rural and 57% (95%CI: 51.6–62.8) in urban areas. Vaccination coverage with at least one dose was 82% (95%CI: 77.3–85.5), 61% (95%CI: 52.0–70.2) in rural and 83% (95%CI: 78.5–87.1) in urban areas.

**Conclusions:**

The Freetown OCV campaign exemplified a timely public health intervention to prevent a cholera outbreak, even if coverage was lower than expected. We hypothesised that vaccination coverage in Freetown was sufficient in providing at least short-term immunity to the population. However, long-term interventions to ensure access to safe water and sanitation are needed.

## Highlights

• Combine door-to-door with fixed or semi-fixed vaccination teams.
• Allow for extended immunization period and self-administered second dose.
• Vaccinate frontline health workers since they are at great risk of exposure.
• Strengthen supervision of the vaccination teams.

## 1 Background

Since the first cholera cases were reported between 1970 and 1971, when the 7^th^ cholera pandemic hit the African continent [1], Sierra Leone experienced large epidemics in 1985, 1994, 1995, and the 1998–2008 period (Supplementary material, Fig. S2). In coastal areas in this part of Africa, cholera spread can increase during the rainy season, between May and October, when flooding is common and water sources are vulnerable to fecal contamination [2], [3]. In 2012, Sierra Leone experienced its largest cholera epidemic in the recent 15 years [4]. From January 2012, and throughout the year, 22,885 cases and 298 deaths were recorded [5]. Most cases were concentrated in the Western Area, which includes the capital Freetown. On 14 August 2017, this region was hit by massive landslides and floods. The landslide epicenter was at Mount Sugarloaf, in the Regent area. It was estimated that 1,141 people died and approximately 5,905 people were displaced [6]. Moreover, because of the landslides and flash floods, sanitary facilities and water pipes were damaged and more than likely contaminated communal water sources.

Most flooded areas contain densely populated slum areas, with challenging access to basic water and sanitation facilities [7]. To avert possible cholera outbreaks following this emergency, the MoHS, supported by the WHO and international partners, including MSF, launched a two-dose pre-emptive vaccination campaign. The target population was defined as any person living in the affected areas and aged ≥1 year old, including pregnant women. The campaign was conducted in the 25 communities in the Freetown metropolitan area most affected by landslides and at the highest risk of cholera. The total target population was 518,104, including both urban (Western Area Urban, with a target population of 495,702 people) and rural areas (Western Area Rural, with a target population of 22,402 people). Two Euvichol^TM^ dose were administered at 2 weeks apart, with the first round administered between the 15^th^ and 20^th^ September 2017 and the second between the 5^th^ and 10^th^ October 2017. Vaccinations were also eventually offered also to those who did not receive them or did not show proof of vaccination during the first round.

The vaccination strategy incorporated a mixture of fixed vaccination posts (i.e. health facilities and schools), semi-mobile vaccination units, and door-to-door vaccination teams.

**Supplementary figure 1:**
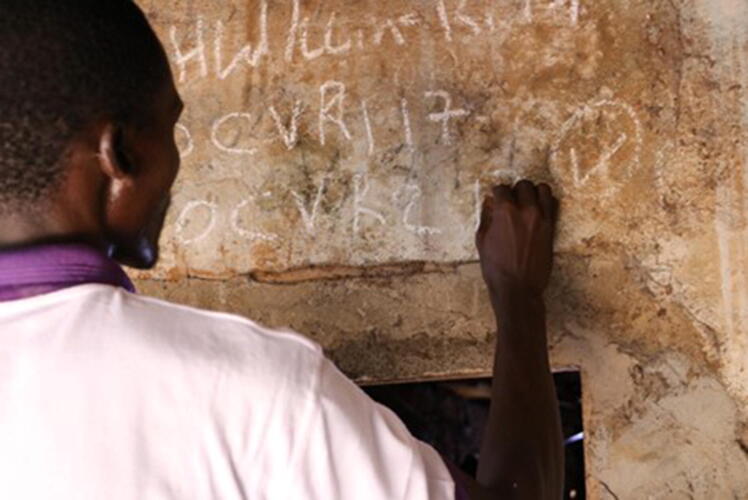
Vaccination teams marking houses visited during the OCV campaign (Photo by WHO/Laura Keenan and Olivia Acland)

**Supplementary figure 2:**
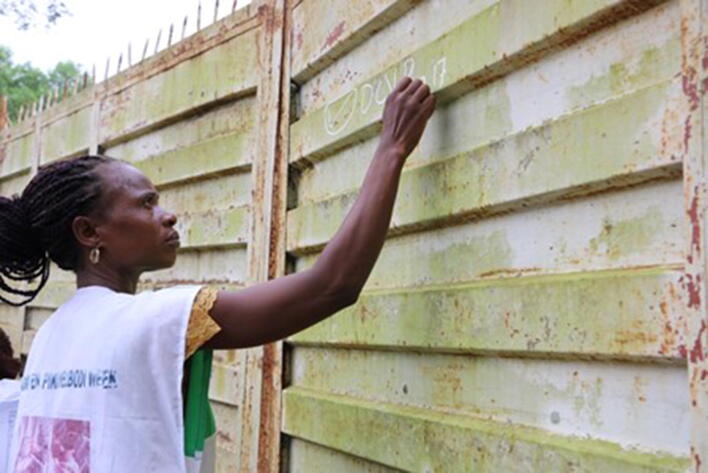
Vaccination teams marking houses visited during the OCV campaign (Photo by WHO/Laura Keenan and Olivia Acland)

For people receiving the vaccine, field teams issued vaccination cards and every visited household was marked at each round as either “fully vaccinated” (all household members were vaccinated), “partially vaccinated” (not all household members were vaccinated), or “not available” (the household had no available beneficiary) (Supplementary material, Fig. S1.a and Fig. S1.b). The team used vaccination cards or individual recall to confirm the vaccination status of people who were to receive their second dose. In addition to vaccination, safe drinking water was provided to affected populations in holding centers, and water quality monitoring was conducted. Multiple partners provided support to different infection prevention and control (IPC) activities, but in a fragmented way and without proper overall coordination, thus diluting the impact of the measure implemented [6]

This was the OCV campaign in Sierra Leone, therefore it was vital to evaluate the campaign and inform future cholera prevention campaigns and public health interventions. Our aim was to estimate vaccination coverage – vis-à-vis the administrative coverage survey reported, by public authorities at the end of the campaign, being 100 % – and also explore reasons for non-vaccination, vaccine acceptability, and identify adverse events following immunization (AEFI)

## 2 Methods

### 2.1 Study population and sample size

We conducted a two-stage stratified cluster survey. All individuals living in one of the 25 communities in the Western Area targeted for vaccination were eligible for inclusion. The target population for the OCV campaign and coverage survey totalled 518,104 or 96 % of the total population in these areas. This represented approximately 34 % of the total population of the Freetown metropolitan area (1,500,234) [8], which constituted Western Area Rural and Western Area Urban.

To estimate vaccine coverage rates according to area type and age groups, the study population was stratified by urban and rural areas and three main age groups: 1–4 years, 5–14 years, and ≥15 years.

Sample size was separately calculated for each stratum and multiplied for each of the three age groups. Based on coverage studies from previous campaigns [9], [10], we assumed a two-dose OCV coverage of 80 %, an alpha error of 5 %, precision of ± 5 %, non-response rate of 10 % and a design effect of 3.0 in urban areas and 2.0 in rural areas. The different design effects between urban and rural were motivated by the hypothesis that vaccinations in the rural areas would be more homogeneous when compared with urban areas. For each age group, we had a sample size of 1,320 people in urban and 880 people in rural areas. For interviews, the total sample size was 3,960 people in urban and 2,640 in rural areas.

From national census data, the average household size was 5.6 persons and the percentage of children aged between 1 and 4 years was approximately 10% [8]. Therefore, we estimated a sample size of 2,357 households in urban and 1,571 households in rural areas on average, to generate the desired number of people in the 1–4 year age group.

Households were assigned to clusters. In urban areas, we selected 86 clusters, with an average of 28 households and totalling 48 people (16 people per each age group) for interview. In rural areas, we selected 69 clusters, with an average of 23 households for an expected total of 39 people (13 people per each age group) for interview. Clusters were chosen based on probability proportional to size (PPS) and the measure of size (MOS) adopted was the number of households. In selected clusters, field teams identified a starting point by selecting a landmark or a main street intersection on the cluster perimeter. The residence closest to that point was deemed the initial household for interview. All subsequent households were identified by moving to the right of the last sampled household. In urban areas, every 5th household was sampled, while in rural areas it was every third household. If no one was available for interview, field teams returned at a different time and/or day.

In each selected household – defined as “a person or group of persons who normally live together, eat from the same pot and recognize a particular person as the head” [8] – field interviewers compiled a list of household members and randomly selected one participant from the three age groups. For people aged < 15 years old, their parent/caretaker answered questionnaires even if the selected person was absent. For people aged ≥15 years or for parents/caretakers, if the selected person was not at home, the interviewer rescheduled a second visit either later that day or a different day.

### 2.2 Sampling frame

The sampling frame was the same as for the national census and based on Enumeration Areas (EAs) – operational geographical units used for census data collection. EAs constituted clusters in our coverage survey. But the rationale followed for the implementation of the campaign and also to allocate vaccination team was that of the catchment area, based and centred on a health facility. At the start of the campaign, each one of the 25 target communities was identified in correspondence to a referral health facility and the portion of the territory served by that facility. For this reason, we had to first fit one sampling frame into the other by disaggregating each catchment area into EAs. The number of clusters allocated to each catchment area was calculated as the percentage of the population targeted during the vaccination campaign in each catchment area over the total targeted population.

### 2.3 Data collection and definitions

Data were collected using standardized questionnaires at face-to-face interviews conducted by trained interviewers. Questionnaires collected information on vaccination house-marking – a chalk mark left on each visited residence during the vaccination campaign; participant socio-demographic characteristics; previous cholera episodes and associated symptomsas certained by recall; living conditions, including latrine type and water source for drinking and cooking; OCV intake; AEFI occurrence and type; potential reasons for non-vaccination and overall vaccine acceptability and perception.

Answers were recorded on smartphone/tablet devices and electronically updated, at the end of each working day on a private server independently managed by WHO using Open Data Kit (ODK). House-marking was assessed by direct observations by field interviewers. In total, 25 teams were employed and each team was composed of two people, a woman and a man. Also, 12 supervisors and six monitors were deployed to oversee and support fieldwork. The coverage survey lasted 12 days, including 3 training days by the MoHS and international partners, and 1 day of piloting. Data were securely downloaded in a Microsoft Excel format. Data cleaning was performed to check for data entry and responses inconsistencies using STATA 13 (Stata Corporation, TX, USA).

### 2.4 Acceptability

OCV acceptability was assessed by direct asking participants how the vaccine impacted their health, how they perceived vaccine taste and the main reasons for taking the vaccine.

### 2.5 Surveillance of adverse events following immunization (AEFI)

We defined an AEFI as any “untoward medical occurrence which follows immunization, and which does not necessarily have a causal relationship with the usage of the vaccine. The adverse event may be any unfavorable or unintended sign, abnormal laboratory finding, symptom or disease” [11]. We asked participants if they had experienced any adverse event following the OCV dose, and recorded symptom/s. Standard WHO AEFI reporting forms documenting adverse events and severity were used for reporting and if needed were used for referral to appropriate case management [12].

### 2.6 Data analysis

Our main outcome was to estimate vaccination coverage as determined by the number of participants who received vaccinations divided by the survey population. We calculated vaccine coverage based on vaccination status as identified by vaccination cards and individual recall. Moreover, vaccine coverage was estimated by age groups (1–4 years, 5–14 years and ≥15 years), different areas (urban or rural), and the number of vaccine doses received (one or two ). Secondary outcomes included reasons for non-vaccination, AEFI, participant living conditions of participants, acceptability and vaccine perception. Data analysis was conducted using STATA 13 (Stata Corporation) whiallowing for complex survey design analysis using sampling weights to account for cluster size differences. The survey design was adopted to calculate point estimates using 95 % CI.

### 2.7 Ethical considerations

The study protocol was reviewed and approved by Sierra Leone Ethics and Scientific Review Committee. This survey fulfilled exemption criteria established by the MSF Ethics Review Board (MSF-ERB) for Intersectional Surveys on vaccination, nutrition, and mortality, and did not require MSF ERB review. The study was conducted with permission from the Medical Director, Operational Center Amsterdam, MSF. Oral informed consent was obtained from all participants. As no personally identifiable information was collected and the study constituted no risk to participants, verbal consent to participate was deemed sufficient. Children <5 years old were represented by parents/guardians whereas participants <15 years old were consented by parents/guardians. No name-related data were collected, thereby eliminating participant identification. Participant privacy, confidentiality, and rights were ensured throughout the study. No compensation or payment was provided for participation.

## 3 Results

The study was conducted between the 21^st^ and 29^th^ October 2017. In total, 3,115 households were visited, of which 106 (3.4 %) were vacant (no one inside) even after a second visit, 31 (1 %) refused to participate and 2,978 (95.6 %) were interviewed. Of interviewed households, 2,961 (99.4 %) provided complete information whereas 17 (0.6 %) had missing information. Overall, 7,189 individuals were interviewed, of which 3,167 (44 %) male and 4,022 (56 %) were female (Table 1).

**Table 1:** Demographic characteristics and living conditions of participants, by type of area. Freetown, Sierra Leone. 2017.

	**RURAL**	**URBAN**	**TOTAL**
**Age** (median)	9.5 yr. IQR: 4 – 24 yr.	10 yr. IQR: 4 – 25 yr.	10 yr. IQR: 4 – 25 yr.
**Sex**			
Male	1,345 (47 %)	1,822 (40 %)	3,167 (44 %)
Female	1,477 (53 %)	2,545 (60 %)	4,022 (56 %)
**Type of sanitation facility (per household)**			
Open defecation	117 (4 %)	135 (3 %)	252 (3 %)
Unimproved facilities	306 (11 %)	899 (21 %)	1,205 (17 %)
Shared	97 (3 %)	125 (3 %)	222 (3 %)
Improved	2,286 (82 %)	3,181 (73 %)	5,467 (77 %)
**Source of drinking water (per household)**			
Surface water	120 (4 %)	20 (0.5 %)	140 (2 %)
Unprotected dug well	383 (14 %)	287 (7 %)	670 (9 %)
Bottled water	564 (20 %)	788 (18 %)	1,352 (19 %)
Protected dug well	907 (32 %)	938 (21.5 %)	1,845 (26 %)
Rain water	24 (1 %)	22 (0.5 %)	46 (1 %)
Tap water	740 (26 %)	2,269 (52 %)	3,009 (42 %)
Other	68 (3 %)	23 (0.5 %)	91 (1 %)
**Source of water used for cooking (per household)**			
Surface water	139 (5 %)	37 (1 %)	176 (2.5 %)
Unprotected dug well	517 (18 %)	525 (12 %)	1,042 (15 %)
Bottled water	50 (2 %)	42 (1 %)	92 (1 %)
Protected dug well	1,085 (39 %)	1,474 (34 %)	2,559 (36 %)
Rain water	37 (1 %)	35 (0.5 %)	72 (1 %)
Tap water	893 (32 %)	2,211 (51 %)	3,104 (43 %)
Other	85 (3 %)	23 (0.5 %)	108 (1.5 %)

### 3.1 Living conditions

Participant living conditions are documented (Table 1) and highlighted the fact that many households used unimproved water sources (surface water, unprotected wells and bottled water) as their main drinking or cooking sources, and had access to shared latrines or unimproved facilities.

### 3.2 OCV coverage

**Fig. 1:**
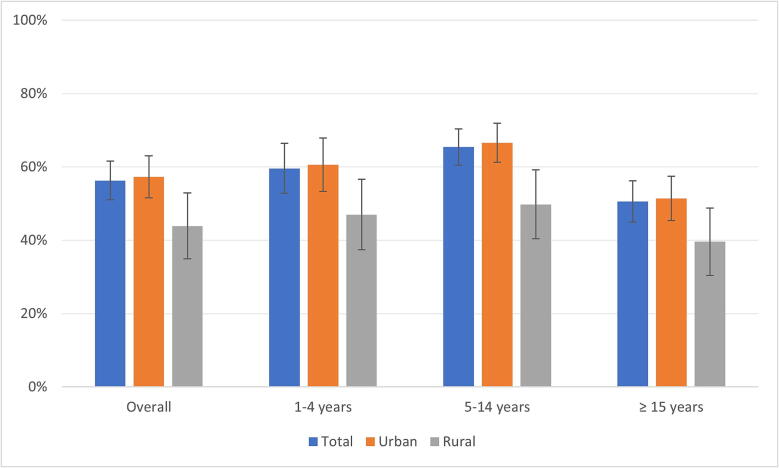
Two-dose vaccination coverage (recall or card), by age group and type of area. Freetown, Sierra Leone. 2017.

Overall, two-dose vaccination coverage, by recall or vaccination card, was 56% (95% CI: 51.0–61.5), 44% (95% CI: 35.2–53.0) in rural and 57% (95% CI: 51.6–62.8) in urban areas (Fig. 1). The two-dose immunisation, by recall or vaccination card, by age group was 60% (95% CI: 52.7–66.1) among children aged 1–4 years, 65% (95% CI: 60.3–70.2) in children aged 5–14 years and 51% (95% CI: 45.0–56.2) in individuals aged ≥15 years old (Supplementary material, Table S2).

**Fig. 2:**
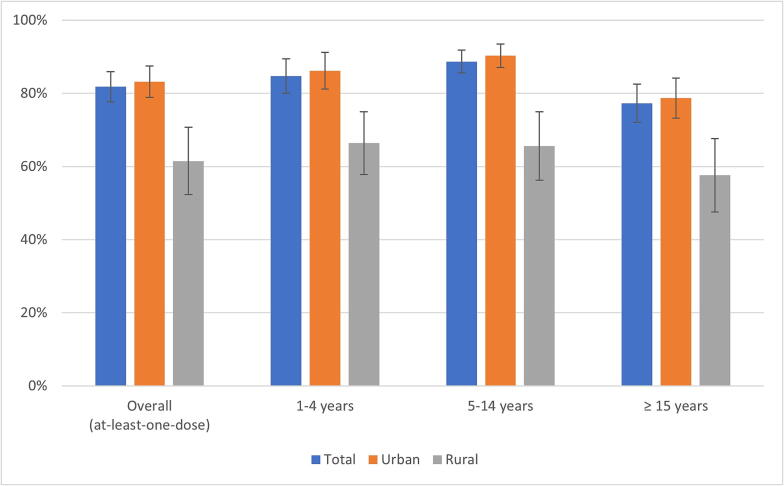
At least one-dose vaccination coverage (recall or card), by age group and type of area. Freetown, Sierra Leone. 2017.

Vaccination coverage with at least one dose, by vaccination card or recall, was 82%; 61% (95% CI: 52.0–70.2) in rural and 83% (95% CI: 78.5–87.1) in urban areas (Fig. 2). When analysing coverage by age group, 85% (95% CI: 79.4–88.8) of children aged 1–4 years received at least one OCV dose when compared with 89% (95% CI: 85.2–91.4) of children aged 5–14 years and 77% (95% CI: 71.7–82.0) of people aged 15 years or more (Supplementary material, Table S2).

### 3.3 Place of vaccination

Overall, 77% (95% CI: 72.5–81.4) of participants received their first dose at home; 71% (95% CI: 65.1–75.6) in rural areas compared with 78% (95% CI: 72.6–82.0) in urban areas (Table 2).

**Table 2:** Place of vaccination, first and second dose. Freetown, Sierra Leone. 2017.

**Location**	**First dose**	**Second dose**
	**N (%)**	**N (%)**
Home	4,161 (77.3)	2854 (77.0)
Health facility	142 (2.0)	96 (1.3)
Outreach site	259 (3.3)	139 (2.4)
School	757 (12.8)	569 (14.7)
Camp	67 (0.3)	61 (0.3)
Market	89 (2.6)	62 (3.2)
Street	40 (0.9)	13 (0.5)
Church	4 (0.1)	2 (0.1)
Office	7 (0.1)	6 (0.1)
Other	35 (0.6)	12 (0.6)

### 3.4 Reason for non-vaccination

For interviewed participants, 18% (95% CI: 14.2–22.3) did not receive an OCV; 38% (95% CI: 29.3–47.7) were in rural and 17% (95% CI: 12.4–21.1) in urban areas. In terms of age group, participants not receiving any vaccination accounted for 15% (95% CI: 10.6–20.0) in children aged 1–4 years, 11% (95% CI: 8.3–14.4) in 5–14 years old and 23% (95% CI: 17.6–27.9) in individuals aged ≥15 years old.

The two most common reasons for not taking any vaccine doses were; 1) participants were not at home during vaccinations (44%) and 2) vaccination teams did not reach his/her house (41%). The two most common reasons for not taking the second vaccine dose were; 1) participants not being at home when vaccination teams called (49%) and 2) vaccination teams did not reach the participant's house (41%).

### 3.5 Adverse events following immunization (AEFI)

In total, 534 AEFIs were reported in 10% (n = 494) of those who received the vaccine. The most reported AEFI symptoms were; fever, diarrhoea and headache (Supplementary material, Table S3). AEFIs were more commonly reported in children aged 1–4 years old and individuals aged ≥ 15 years old when compared with children aged 5–14 years old. AEFIs were not serious and required no medical referral.

### 3.6 Acceptability and perception

Of those participants who were asked if they previously had cholera, only 4% (95% CI: 3.0–4.6) reported having already contracted the disease. Of these, 85% received at least one vaccine dose. The majority of vaccinated respondents, 90% (95% CI: 88.5–92.0) had a positive opinion on the vaccine, with 77% (95% CI: 73.1–81.4) reporting they were motivated to have the vaccination as they had prior knowledge of cholera and its risks.

## 4 Discussion

In Freetown, vaccination coverage (recall or vaccination card) with at least one dose was 82% (95% CI: 77.3–85.5) and 56% (95% CI: 51.0–61.5) for a two-dose vaccination. It was striking that such coverage had occurred during the campaign; approximately 15 days had elapsed since the request from the International Coordinating Group (ICG) on Vaccine Provision  (29^th^ August 2017) to the first vaccination rounds (15^th^ September 2017). Our survey data were similar to other OCV campaigns in metropolitan and densely populated areas [13], [14], [15], [16], [17], with coverage likely providing short-term protection for most at-risk populations [18], [19]. At the time of writing, in February 2023, no major cholera outbreaks had been reported in the Freetown areas where the campaign was implemented [20]. Other studies reported that the herd effect started at 28% vaccine coverage [21] and therefore, it was likely our vaccine campaign may have protected neighbouring un-vaccinated communities [22], [23], [24], [25]. Moreover, vaccinations may have acted as a booster for people previously exposed to cholera, further increasing protection [26].

**Supplementary figure 3:**
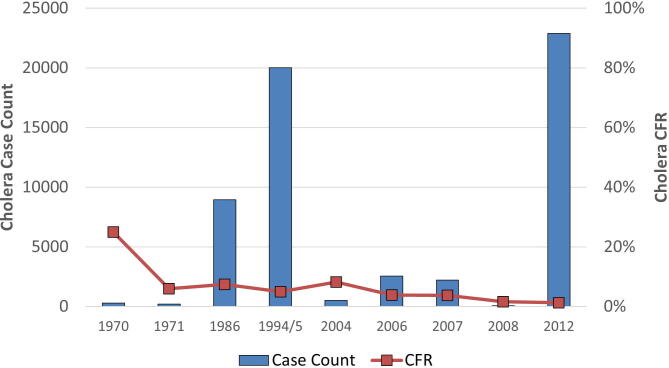
Reported Cholera Case Count and Case Fatality Ratio (CFR) in Sierra Leone (1970-2012)

While prompt responses through our vaccination campaign may have provided some cholera protection to the population, the fact the campaign was organised in such a short time may have caused some operational weaknesses. Coverage in Freetown deviated considerably from vaccination campaign objectives, which were set at 95% coverage for the two-dose vaccination by the MoHS Ministry of Health, and 100% coverage in administrative reports. However, our Freetown percentages are commonly observed in large urban vaccination campaigns [9], [13], and were possibly due to several factors. Firstly, the target population was initially calculated using health facility catchment areas, an operational definition that differed from the national census and other governmental institutions. Consequently, the target population, as calculated at the start of the campaign, was probably overestimated and misaligned with populations in targeted areas (Supplementary material, Figure S3). Thus, the low coverage rate may be partially explained by the fact that some people, vaccinated during the campaign, did not live in targeted areas and could not be accounted for during the coverage survey. A second factor is related to high mobility in the population. Overall, the dropout rate was 31%, but  slightly higher among urban populations (31%) when compared with rural populations (29%), and higher in those aged ≥ 15 years (34%) when compared with children aged 5–14 (26%) and 1–4 years old (30%). Consistent with other studies  [9], [10], [13], [27], individuals aged ≥ 15 years, particularly men, had lower coverage rates than other age groups and were the most challenging to vaccinate. This cohort may have been more mobile and therefore harder to reach. Such challenges could be mitigated by permitting different vaccination strategies to complement and strengthen OCV campaign strategies [28]; flexible (i.e., night-time immunisation services) and/or extended vaccination schedules (i.e., additional vaccine stocks at health centres), alternative vaccine uptake (i.e., self-administered second doses), targeted communications and a wider field vaccination site network (i.e., workplace vaccinations).

When reviewing vaccination coverage in each community (Supplementary material, Table S1), for at least one and two doses, rural areas had a lower coverage when compared with urban areas. Moreover, some communities, despite having a high coverage for at least one dose, had very low coverage for two doses. This suggested that during each round, different people may have received the vaccination. Indeed, even though the vaccination strategy in Freetown included a mixture of door-to-door and fixed or semi-mobile teams, these approaches were not well balanced and the campaign strategy lost some effectiveness. For example, not enough mobile teams were assigned to areas which may have benefited more from a more flexible and *ad hoc* approach. Door-to-door approaches were problematic in rural areas where boundaries between different catchment areas were ill-defined, leaving entire sections completely “missed” by vaccination teams.

Following immunisation, no severe adverse events were reported. In general, AEFIs did not represent a major concern as their patterns reflected other OCV campaigns [29]. Finally, vaccine acceptability and perceptions were largely positive, probably due to previous knowledge/experiences of cholera and its dangers.

## 5 Strengths and limitations

Major study strengths included its large sample size and stratification by age groups and area type. Independently sampling specific subpopulations provided better accuracy and allowed for proper comparisons in the study population. The main limitation was that the national census sampling frame, the sampling adopted in this study, failed to perfectly overlap with catchment areas during the vaccination campaign, mainly because the latter was an *ad hoc* operational geographical unit conducting vaccinations. Another limitation, which commonly affects post-campaign coverage surveys [34], was recall bias and vaccination card loss. These issues may have been mitigated by conducting the survey at the end of the vaccination campaign, thereby reducing possible bias.

Finally, in selected clusters, interviewers identified landmarks or main street starting points on cluster perimeters. This approach may have cause bias and possible coverage overestimations given that households near main street intersection or landmarks were most accessible to vaccinating teams during the campaign.

## 6 Conclusion

This OCV campaign in Freetown was a good example of a timely public health anti-cholera intervention, even if coverage was lower than expected. Drawing from other studies and previous vaccination campaigns [18], [30], [31], [32], [33], we hypothesised that vaccination coverage in Freetown likely provided at least short-term immunity to the population. However, long-term strategies ensuring access to safe water and sanitation are needed. Our study data become more relevant when we consider the campaign was organised in an emergency context, in a geographically-challenging environment and with limited preparation time. Future urban and rural area campaigns will benefit from the lessons outlined in this study.

## Declaration of Competing Interest

The authors declare that they have no known competing financial interests or personal relationships that could have appeared to influence the work reported in this paper.

## Data Availability

Data will be made available on request.
