# Supplementary material for: Coverage survey and lessons learned from a pre-emptive cholera vaccination campaign in urban and rural communities affected by landslides and floods in Freetown Sierra Leone
Source: Vaccine. 2023 Mar 31;41(14):2397–403. doi: 10.1016/j.vaccine.2023.01.026 (PMC10102719; doi:10.1016/j.vaccine.2023.01.026)
Supplement: Supplementary data 1 [file mmc1.docx]

**Supplementary material**

**Table S1 – Coverage rate for at least one-dose and two-doses, by community**

| **Community** | **Type** | **Coverage** | | | |
| --- | --- | --- | --- | --- | --- |
|  |  | **At least one-dose** | | **Two-doses** | |
|  |  | % | (95% CI; deff) | % | (95% CI; deff) |
| Charlotte | Rural | 46 | (15.6 -79.2; 8.7) | 34 | (9.7-71.8; 8.7) |
| Leicester | Rural | 53 | (35.8-69.3; 4.8) | 37 | (23.7-53.3; 3.9) |
| Regent | Rural | 80 | (74.1-84.7; 1.8) | 65 | (58.0-71.7; 2.2) |
| George Brook | Urban | 77 | (65.8-86; 11.4) | 46 | (33.5-58.8; 12.9) |
| Grey Bush | Urban | 83 | (75.5-87.6; 1.4) | 66 | (53.3-76.5; 3.4) |
| Hill Station | Urban | 74 | (58.3-85.5; 2.6) | 43 | (29.5-57.7; 2.3) |
| Iscon | Urban | 89 | (84.4-92.9; 1.7) | 74 | (69.6-78.9; 1.0) |
| Jenner Wright | Urban | 92 | (83.6-96.0; 4.5) | 77 | (67.0-85.1; 4.6) |
| King Harmann Road | Urban | 63 | (32.8-85.2; 44.7) | 44 | (14.9-77.5; 68.3) |
| Kingtom | Urban | 92 | (88.9-94.8; 0.7) | 77 | (46.9-92.5; 17.4) |
| Konikay | Urban | 63 | (12.6-95.2; 96.3) | 24 | (6.5-59.2; 28.8) |
| Kroobay | Urban | 64 | (39.2-83.5; 24.6) | 38 | (18.7-63.0; 24.1) |
| Lumley | Urban | 95 | (89.2-97.7; 2.8) | 63 | (55.5-70.4; 2.1) |
| Mabella | Urban | 99 | (94.3-99.9; 1.7) | 83 | (58.4-94.6; 11.7) |
| Macauley St. (Mountain Cut) | Urban | 91 | (73.3-97.4; 28.2) | 76 | (63.7-84.5; 12.0) |
| Malama | Urban | 90 | (77.9-96.2; 3.9) | 37 | (3.6-90.5; 83.0) |
| Moyeba | Urban | 78 | (54.8-91.5; 29.9) | 46 | (17.9-77.1; 68.4) |
| ODCH – Moa Wharf | Urban | 80 | (72.0-86.1; 2.6) | 51 | (39.0-62.8; 5.0) |
| PAYCY | Urban | 88 | (78.9-93.3; 1.8) | 32 | (11.3-62.8; 13.6) |
| Pentagon | Urban | 89 | (87.7-90.6; 0.1) | 12 | (3.0-36.1; 7.3) |
| SLIMS | Urban | 98 | (94.7-99.5; 1.2) | 74 | (48.9-90.0; 11.6) |
| Stella Maries | Urban | 82 | (71.6-88.7; 2.2) | 60 | (36.0-80.5; 10.7) |
| Susan’s Bay | Urban | 99 | (97.4-99.8; 0.6) | 79 | (54.6-92.4; 12.0) |
| UMC Urban Center | Urban | 88 | (71.8-95.8; 2.5) | 45 | (28.9-62.8; 2.5) |

**Table S2 – Vaccination coverage for two-doses and at least one-dose, by age group and type of cluster. Freetown, Sierra Leone. 2017**

|  | **Two-doses** | | | **At least one-dose** | | |
| --- | --- | --- | --- | --- | --- | --- |
|  | *Rural* | *Urban* | *Overall* | *Rural* | *Urban* | *Overall* |
| **1 – 4 years** | 47%  (95%CI: 37.4-56.6; deff=0.9) | 61%  (95%CI: 53.3-67.9; deff=6.7) | 60%  (95%CI: 52.7-66.1; deff=6.2) | 66%  (95%CI: 57.8-75.0; deff=0.8) | 86%  (95%CI: 81.2-91.3; deff=6.5) | 85%  (95%CI: 79.4-88.8; deff=5.6) |
| **5 – 14 years** | 50%  (95%CI: 40.4-59.2; deff=1.2) | 67%  (95%CI: 61.3-71.9; deff=5.9) | 65%  (95%CI: 60.3-70.2; deff=5.5) | 66%  (95%CI: 56.2-74.9; deff=1.3) | 90%  (95%CI: 87.1-93.5; deff=5.6) | 89%  (95%CI: 85.2-91.4; deff=4.8) |
| **≥ 15 years** | 40%  (95%CI: 30.4-48.8; deff=2.3) | 51%  (95%CI: 45.4-57.4; deff=13.4) | 51%  (95%CI: 45.0-56.2; deff=12.7) | 58%  (95%CI: 47.6-67.6; deff=2.7) | 79%  (95%CI: 73.2-84.1; deff=16.5) | 77%  (95%CI: 71.7-82.0; deff=15.0) |
| **Total** | 44%  (95%CI: 35.2-53.0; deff=4.1) | 57%  (95%CI: 51.6-62.8; deff=22.1) | 56%  (95%CI: 51.0-61.5;  deff=20.7) | 61%  (95%CI: 52.0-70.2; deff=4.5) | 83%  (95%CI: 78.5-87.1; deff=22.9) | 82%  (95%CI: 77.3-85.5; deff=20.4) |

**Table S3 – Type of AEFI among people who received at least one dose of OCV, by dose**

| **AEFI Symptom reported** | **After First dose**  **N (%)** | **After Second dose**  **N (%)** |
| --- | --- | --- |
| Nausea | 13 (3%) | 6 (5%) |
| Diarrhoea | 88 (21%) | 33 (28%) |
| Fever | 131 (32%) | 41 (35%) |
| Vomiting | 38 (9%) | 3 (3%) |
| Abdominal pain | 38 (9%) | 3 (3%) |
| Headache | 38 (9%) | 7 (6%) |
| Rash | 37 (9%) | 18 (15%) |
| Other | 35 (8%) | 5 (4%) |
| Total | 418 (100%) | 116 (100%) |

**Figure S1.a – Vaccination teams marking houses visited during the OCV campaign (**Photo by WHO/Laura Keenan and Olivia Acland)

**Figure S1.b – Vaccination teams marking houses visited during the OCV campaign (**Photo by WHO/Laura Keenan and Olivia Acland)

**Figure S2 – Reported Cholera Case Count and Case Fatality Ratio (CFR) in Sierra Leone (1970-2012)**
